# Increasing HIV and Decreasing Syphilis Prevalence in a Context of Persistently High Unprotected Anal Intercourse, Six Consecutive Annual Surveys among Men Who Have Sex with Men in Guangzhou, China, 2008 to 2013

**DOI:** 10.1371/journal.pone.0103136

**Published:** 2014-07-25

**Authors:** Fei Zhong, Boheng Liang, Huifang Xu, Weibin Cheng, Lirui Fan, Zhigang Han, Caiyun Liang, Kai Gao, Huixia Mai, Faju Qin, Jinkou Zhao, Li Ling

**Affiliations:** 1 School of Public Health, Sun Yat-sen University, Guangzhou, China; 2 Department of HIV/AIDS Control and Prevention, Guangzhou Center for Disease Control and Prevention, Guangzhou, China; 3 Sun Yat-sen Center for Migrant Health Policy, Sun Yat-sen University, Guangzhou, China; 4 The Global Fund to fight AIDS, Tuberculosis and Malaria, Geneva, Switzerland; Catalan Health Institute, Spain

## Abstract

**Introduction:**

Previous studies have reported a possibly increasing HIV prevalence among men who have sex with men (MSM) in China. However there have been limited systematic analyses of existing surveillance data to learn the trend of HIV prevalence and factors driving the trend. The aims of this study were to examine the trend of HIV prevalence among MSM in Guangzhou and to explore the role of unprotected anal intercourse (UAI) in the trend.

**Methods:**

Snow-ball sampling was applied in the subject recruitment for the annual serological and behavioral surveys among MSM from 2008 to 2013. Data collected in the behavioral survey include demographic information, HIV related sexual behavior with men and women, access to HIV prevention services, and symptoms of sexually transmitted infections. Chi-square test was used to analyze the trend of HIV prevalence. Multivariate logistic regression was conducted to test the factors associated with HIV infection.

**Results:**

HIV prevalence increased significantly from 5.0% in 2008 to 11.4% in 2013 while syphilis prevalence decreased from 17.4% to 3.3% in the same period. UAI rates were high and stable in every single year, ranging from 54.5% to 62.0%. Those who were having UAI (OR = 1.80, 95% confidence interval (CI): 1.26–2.58), being migrants, having more than 10 partners, and infected with syphilis had higher risk for HIV infection.

**Conclusions:**

HIV epidemic is expanding in Guangzhou. The persistently high UAI may have played a major role in the increasing trend of HIV prevalence. Targeted prevention program should be conducted among MSM who are migrants, low educational level, syphilis infected, or having multiple partners to encourage HIV test and change UAI behavior. The general high UAI calls for tailored intervention program to promote healthy culture and form a safe sex social norm in the MSM community.

## Introduction

During the past decade, the HIV epidemiology has changed in China, from predominantly driven by injection drug use and unsafe plasma collection to by unprotected sex [1]. Among reported HIV/AIDS cases in China, the proportion of men who have sex with men (MSM) increased from 7.3% in 2005, to 11.0% in 2007 and 17.4% in 2011 [2]. Unprotected sex between men has become one of the major HIV transmission modes in China, accounting for 29.4% of estimated annual new HIV infection in China in 2011 [2].

Guangzhou, the capital of Guangdong province and one of the largest cities located in southern China, is remarkable for its rapid economic growth during the past three decades, thanks to the open-door policy since 1978 [3]. The open-door policy brought the opportunities not only for economic development, but for multiple culture environments. Guangzhou gradually became socially tolerant city, which attracts MSM from all over the country and other surrounding Asian cities. During the past decade, lots of gay cruising areas and partner hunting ways emerged, such traditional ones as gay bars, sauna, tea house, parks, public toilets and clubs, and modern ones as gay website, internet chat-room, instant chatting groups, and mobile networking. In addition, highways and high speedy trains make partner hunting very easy for MSM between Guangzhou and its neighboring cities. Easy mobility, together with advanced modern networking tools, incubates spreading of sexually transmitted diseases including HIV when sex is unprotected.

Unprotected anal intercourse (UAI) might have played a key role in the spread of HIV among MSM in Guangzhou. The relationship between UAI and HIV infection and sexually transmitted infections (STIs) is biologically plausible and approved by numerous studies in the United States and Australia [4]–[6]. Reports in different Chinese cities indicated that UAI has increased or maintained at a high level, despite a high level of knowledge about HIV/AIDS, since early 2000s when most of the studies were conducted [7].

Previous surveys have reported a possibly increasing average HIV prevalence among MSM in China, from 0.9% in 2003, 1.3% in 2004, 3.0% in 2006 and 5.0% in 2008 [8]. Increased HIV prevalence has been reported in big Chinese cities. In Beijing, HIV prevalence of MSM increased from 0.4% in 2004 to 5.8% in 2006, and 8.0% in 2009 [9], [10]. In Harbin, the prevalence of HIV increased from 1.0% in 2006 to 7.5% in 2010 [11]. The HIV prevalence was reported to be increasing in some medium-sized cities like Hangzhou (from 1.8% in 2006 to 8.3% in 2009) [12], Jinan (from 0.05% in 2007 to 3.1% in 2008) [13], Suzhou (from 7.1% in 2008 to 8.2% in 2012) [14], [15]. This increase is in the context of a regional increase in Asia such as from 17.3% in 2003 to 28.3% in 2005 in Bangkok [16], from 2.5% in 2002 to 8.0% in 2007 in Jakarta [17], from 9.4% in 2006 to 20% in 2010 in Hanoi [18].

There might be an increase in HIV prevalence in Guangzhou. However, there have been limited systematic analyses of existing surveillance data to examine the trend of HIV prevalence and factors driving the trend [11]. Taking advantage of being part of 61 cities' survey among MSM in China organized by Chinese Center for Disease Control and Prevention (China CDC) [19], Guangzhou started the annual biological and behavioral surveys among MSM since 2008 using an identical survey protocol and the same questionnaire, implemented by the same group of interviewers at the same survey site.

In the present study, we examined the trend of HIV prevalence and behaviors using annual biological and behavioral surveys from 2008 to 2013. We also explored the role of UAI in the trend of HIV prevalence.

## Materials and Methods

### Participants and recruitment

Subjects included in the surveys were men who had anal or oral sex with men in the last 12 months, at least 18 years old and had lived in Guangzhou for at least 6 months prior to the survey year.

Snow-ball sampling was applied in the subject recruitment. An incentive, including a gift (worth about 2 US dollars) and 20 Chinese Yuan cash (approximately 3 US dollars), was given for participation in the questionnaire survey and the provision of 5 ml blood for serological testing. Pre- and post-test counseling services were provided to each of subjects participating in the study. Referral services were provided to HIV or syphilis positive cases.

The sample size was estimated based on the following formula [20]:
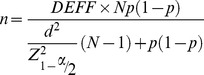



The MSM population size (*N*) in Guangzhou was estimated to be about 35,000 (Personal communication, Huifang Xu, unpublished data based on capture-recapture exercise in 2008). HIV prevalence among MSM (*p*) was 5.2% in 2008 [21], confidence limit (*d*) of 5%, a design effect (*DEFF*) of 2, and an alpha (*α*) of 0.05. Given these conditions, the required smallest sample size would be 152. The sample size was increased to 400 to allow for greater representativeness and possible refusals during the recruitment.

The study protocol was developed by Guangzhou CDC and approved by its Ethics Committee. All subjects provided written informed consents.

### Data collection

After written informed consent obtained from each of the eligible subjects, 5 ml intravenous blood was drawn prior to the questionnaire interview. Face to face questionnaire interviews were conducted by the same interviewers using an identical structured questionnaire in a private room at the voluntary counseling and testing (VCT) clinic of Guangzhou CDC.

Data collected in the questionnaire include demographic information, knowledge and attitudes towards HIV or AIDS, access to HIV prevention services, and sexual behavior with men and women in the past 6 months, and symptoms of STIs in the past 12 months. The questionnaire interviews were administered anonymously but such contact information as mobile phone numbers, QQ (Instant messaging software) account or email address were collected to inform the laboratory test results and referral services when necessary.

HIV screening was conducted by using two enzyme-linked immunoassays (ELISAs; Diagnostic Kit for Antibody to Human Immunodeficiency Virus, BioMérieux, Boxtel, The Netherlands, Beijing BGI-GBI Biotech, Beijing, China). If the result of one ELISA was positive, a Western Blot (WB) test was conducted for confirmation (WB;MP Biomedicals Asia Pacific Pte Ltd, Singapore). Syphilis screening was performed by rapid plasma regain (RPR; Shanghai Kehua Bioengineering Co. Ltd, Shanghai, China) to test anticardiolipin. Specimens testing positive by RPR were confirmed by the treponema pallidum particle agglutination test (TPPA; Livzon Group Reagent Factory, Zhuhai, China) to test treponema pallidum antibody. Specimen which is positive in both RPR and TPPA can be diagnosed to be syphilis infection.

### Data analysis

Although the sampling method of survey was a non-probability sample, the sample size was considered large enough to be representative of the population. In this study, UAI was defined as failure to consistently use a condom during the past 6 months when having anal sex. Having no anal sex reported was considered to be safe. UAI was categorized as ‘No’ if there was no anal intercourse or a condom was used every time, and ‘Yes’ if a condom was not used all the time. Unprotected vaginal intercourse (UVI) was categorized in the same method. Coverage of prevention services was defined as receiving any service including condom distribution, lubricant distribution, peer education, STI diagnosis or treatment, HIV counseling or testing, or AIDS/STI educational materials in the past 12 months.

Data were double entered and cleaned using EpiData (version 3.1, Denmark). SPSS (version 17.0, LEAD Technologies Inc.) was used to perform Pearson chi-square test, linear-by-linear association chi-square test, likelihood ratio chi-square test, and multivariate logistic regression analysis. The multivariate forward stepwise logistic regression was conducted to test the factors associated with HIV infection. The independent variable input in the regression model of HIV infection included age, marital status, Hukou (registered permanent residence), education level, ethnicity, monthly income, age at first sex with male, years of being MSM, sexual orientation, venues for meeting partners, UAI, UVI, HIV testing history, preventive intervention services received, STI symptoms in the past 12 months, syphilis infection, and with survey year as dummy variable. All statistical significance test results are reported as p-values, where less than 0.05 was used to define significance.

## Results

From 2008 to 2013, 379, 385, 405, 400, 401 and 633 subjects were recruited respectively during April to July each year. The demographic characteristics and key behaviors of the participants are shown in Table 1. From 2008 to 2013, along with the development of economy and culture in China, the increasing trends were found in some demographic data, including increased proportions of being single, having higher educational level and income, and being internet surfers for meeting partners. There was an increase in the rates of HIV testing and coverage of HIV intervention during the past 12 months from 2008 to 2013. In the meantime, the proportion of subjects, who reported STI symptoms during the past 12 months, with multiple partners, or having UVI in the past 6 months, decreased statistically. Syphilis prevalence decreased from 17.4% in 2008 to 3.3% in 2013 during the same period of time. However, across the six years, the most commonly reported risk behavior remained stable, the percentage of UAI still range 54.5% to 62.0% (p>0.05) (Table 1).

**Table 1:** Demographic characteristics and key behaviors of MSM in Guangzhou, China, 2008–2013.

Characteristics	2008 (N = 379) n (%)	2009 (N = 385) n (%)	2010 (N = 405) n (%)	2011 (N = 400) n (%)	2012 (N = 401) n (%)	2013 (N = 633) n (%)	?^2^ value	*p* a
Age (years)							22.80	0.299
<20	14 (3.7)	10 (2.6)	13 (3.2)	15 (3.8)	9 (2.2)	15 (2.4)		
20–29	217 (57.3)	227 (59.0)	245 (60.5)	250 (62.5)	228 (56.9)	385 (60.8)		
30–39	111 (29.3)	113 (29.4)	120 (29.6)	101 (25.3)	128 (31.9)	174 (27.5)		
40–49	35 (9.2)	27 (7.0)	23 (5.7)	25 (6.3)	24 (6.0)	49 (7.7)		
≥50	2 (0.5)	8 (2.1)	4 (1.0)	9 (2.3)	12 (3.0)	10 (1.6)		
Marital status							40.73	<0.001
Married	79 (20.8)	69 (17.9)	74 (18.3)	89 (22.3)	69 (17.2)	76 (12.0)		
Cohabiting with male partner	67 (17.7)	69 (17.9)	62 (15.3)	46 (11.5)	63 (15.7)	104 (16.4)		
Single	214 (54.5)	232 (60.3)	256 (63.2)	260 (65.0)	258 (64.3)	430 (67.9)		
Divorced or widowed	19 (5.0)	15 (3.9)	13 (3.2)	5 (1.3)	11 (2.7)	23 (3.6)		
Hukou (registered permanent residence)							57.46	<0.001
Guangzhou	105 (27.7)	122 (31.7)	122 (30.1)	115 (28.8)	134 (33.4)	235 (37.1)		
Other city in Guangdong province	85 (22.4)	84 (21.8)	90 (22.2)	67 (16.8)	94 (23.4)	186 (29.4)		
Outside Guangdong province	189 (49.9)	179 (46.5)	193 (47.7)	218 (54.5)	173 (43.1)	212 (33.5)		
Han nationality	362 (95.5)	369 (95.8)	382 (94.3)	381 (95.3)	389 (97.0)	614 (97.0)	6.37	0.272
Education level							64.16	<0.001^b^
Junior high school or lower	72 (19.0)	47 (12.2)	66 (16.3)	53 (13.3)	49 (12.2)	35 (5.5)		
Senior high school	111 (29.3)	99 (25.7)	115 (28.4)	140 (35.0)	103 (25.7)	110 (17.4)		
College or higher	196 (51.7)	239 (62.1)	224 (55.3)	207 (51.8)	249 (62.1)	488 (77.1)		
Monthly income (CNY)							131.60	<0.001^b^
0	49 (12.9)	54 (14.0)	46 (11.4)	39 (9.8)	56 (14.0)	84 (13.3)		
≤2000	125 (33.0)	88 (22.8)	99 (24.5)	65 (16.3)	27 (6.7)	47 (7.4)		
2001–3000	85 (22.4)	95 (24.7)	94 (23.2)	112 (28.0)	78 (19.5)	98 (15.5)		
3001–4000	60 (15.8)	60 (15.6)	69 (17.0)	86 (21.5)	72 (18.0)	112 (17.7)		
>4000	60 (15.8)	88 (22.9)	97 (24.0)	98 (24.5)	168 (41.9)	292 (46.1)		
Self-perceived sexual orientation							26.23	0.003
Homosexual	232 (61.2)	247 (64.2)	264 (65.2)	260 (65.0)	247 (61.6)	451 (71.2)		
Bisexual	106 (28.0)	109 (28.3)	117 (28.9)	120 (30.0)	119 (29.7)	148 (23.4)		
Uncertain	41 (10.8)	29 (7.5)	24 (5.9)	20 (5.0)	35 (8.7)	34 (5.4)		
Venues for meeting partners							170.40	<0.001
Bar, disco, tea house or club	28 (7.4)	31 (8.1)	25 (6.2)	27 (6.8)	22 (5.5)	22 (3.5)		
Bath house, sauna or massage	25 (6.6)	15 (3.9)	14 (3.5)	9 (2.3)	6 (1.5)	8 (1.3)		
Park, public toilet	57 (15.0)	25 (6.5)	24 (5.9)	18 (4.5)	40 (10.0)	5 (0.8)		
Internet	247 (65.2)	273 (70.9)	298 (73.6)	309 (77.3)	282 (70.3)	564 (89.1)		
Other	22 (5.8)	41 (10.6)	44 (10.9)	37 (9.3)	51 (12.7)	34 (5.4)		
Age of first sex with male (years)							1.97	0.161^b^
<19	66 (17.4)	88 (22.9)	63 (15.6)	62 (15.5)	70 (17.5)	118 (18.6)		
19–24	195 (51.5)	178 (46.2)	181 (44.7)	179 (44.8)	192 (48.0)	364 (57.5)		
≥25	118 (31.1)	119 (30.9)	161 (39.8)	159 (39.8)	138 (34.5)	151 (23.9)		
Years of being MSM							7.70	0.006^b^
≤1	24 (6.3)	47 (12.2)	38 (9.4)	24 (6.0)	37 (9.2)	44 (7.0)		
1.1–3.0	82 (21.6)	100 (26.0)	99 (24.4)	99 (24.8)	83 (20.7)	139 (22.0)		
3.1–5.0	86 (22.7)	74 (19.2)	94 (23.2)	112 (28.0)	80 (20.0)	105 (16.6)		
5.1–10.0	124 (32.7)	115 (29.9)	130 (32.1)	111 (27.8)	128 (31.9)	226 (35.7)		
>10	63 (16.6)	49 (12.7)	44 (10.9)	54 (13.5)	73 (18.2)	119 (18.8)		
Have anal sex with male in the past 6 months	336 (88.7)	348 (90.4)	360 (88.9)	369 (92.3)	363 (90.5)	574 (90.7)	3.95	0.556
Number of male partners in the past 6 months							7.45	0.006^b^
1	94 (28.0)	123 (35.3)	121 (33.6)	123 (33.3)	136 (37.5)	184 (32.2)		
2	80 (23.8)	92 (26.4)	96 (26.7)	98 (26.6)	97 (26.7)	159 (27.8)		
3	69 (20.5)	66 (19.0)	63 (17.5)	66 (17.9)	72 (19.8)	112 (19.6)		
4–9	69 (20.5)	38 (10.9)	60 (16.7)	68 (18.4)	40 (11.1)	105 (18.4)		
≥10	24 (7.1)	29 (8.3)	20 (5.6)	14 (3.8)	18 (5.0)	12 (2.1)		
UAI^c^	233 (61.5)	210 (54.5)	240 (59.3)	248 (62.0)	227 (56.6)	361 (57.0)	7.10	0.213
UVI^c^	58 (15.3)	49 (12.7)	64 (15.8)	52 (13.0)	60 (15.0)	53 (8.4)	18.05	0.003
HIV test in the past 12 months	62 (16.4)	274 (71.2)	178 (44.0)	177 (44.7)	198 (49.7)	338 (53.4)	247.15	<0.001
Coverage of HIV intervention	287 (75.7)	338 (87.8)	301 (74.3)	283 (70.8)	334 (83.3)	538 (85.0)	60.94	<0.001
STI symptoms in the past 12 months^c^	85 (22.4)	85 (22.1)	61 (15.1)	64 (16.0)	57 (14.2)	87 (13.7)	23.39	<0.001
Syphilis infection	66 (17.4)	32 (8.3)	28 (6.9)	25 (6.2)	36 (9.0)	21 (3.3)	67.45	<0.001

a Pearson chi-square test.

b Linear-by-linear association chi-square test.

c UAI, unprotected anal intercourse; UVI, unprotected vaginal intercourse; STI, sexually transmitted infection.

HIV prevalence increased significantly from 5.0% in 2008 to 11.4% in 2013 (p<0.001). The increasing trend of HIV prevalence was also found among most of subgroups (Table 2). While in some groups, the rates have been stable over six years. HIV prevalence did not increase significantly in those who cohabited with male partners, having educational level of junior high school or lower, having monthly income lower than 2000 Chinese Yuan, meeting sex partners in bar or bath house, having 4 or more male partners, and syphilis infected, and prevalence among them was higher than average rate in each single year. In another aspect, HIV prevalence did not increase in subgroups those who having income higher than 4000, having less than 2 partners, and having no UAI in the past 6 months, and prevalence in these groups was lower than average rate.

**Table 2:** HIV prevalence by demographic characteristics and sexual behaviors among MSM in Guangzhou, 2008–2013.

Characteristics	2008 % (n)	2009 % (n)	2010 % (n)	2011 % (n)	2012 % (n)	2013 % (n)	?^2^ value	*p* a
Overall	5.0 (19)	3.9 (15)	7.7 (31)	9.3 (37)	10.0 (40)	11.4 (72)	25.42	<0.001
Age (years)								
<20	0.0 (0)	0.0 (0)	0.0 (0)	6.7 (1)	0.0 (0)	20.0 (3)	8.98	0.110^b^
20–29	4.1 (9)	3.5 (8)	8.6 (21)	10.0 (25)	9.2 (21)	12.2 (47)	20.31	0.001
30–39	8.1 (9)	5.3 (6)	7.5 (9)	8.9 (9)	10.9 (14)	8.0 (14)	2.69	0.748
40–49	2.9 (1)	3.7 (1)	4.3 (1)	4.0 (1)	20.8 (5)	10.2 (5)	7.75	0.170^b^
≥50	0.0 (0)	0.0 (0)	0.0 (0)	11.1 (1)	0.0 (0)	30.0 (3)	8.50	0.131^b^
Marital status								
Married	5.1 (4)	0.0 (0)	5.4 (4)	7.9 (7)	14.5 (10)	11.8 (9)	13.76	0.017
Cohabiting with male partner	9.0 (6)	7.2 (5)	4.8 (3)	13.0 (6)	17.5 (11)	14.4 (15)	7.68	0.175
Single	3.7 (8)	3.0 (7)	8.2 (21)	9.2 (24)	6.2 (16)	10.5 (45)	18.79	0.002
Divorced or widowed	5.3 (1)	20.0 (3)	23.1 (3)	0.0 (0)	27.3 (3)	13.0 (3)	5.46	0.363^b^
Hukou (registered permanent residence)								
Guangzhou	1.0 (1)	0.8 (1)	4.9 (6)	5.2 (6)	4.5 (6)	8.1 (19)	13.55	0.019
Other city in Guangdong province	5.9 (5)	0.0 (0)	8.9 (8)	10.4 (7)	9.6 (9)	10.2 (19)	10.11	0.072
Outside Guangdong province	6.9 (13)	7.8 (14)	8.8 (17)	11.0 (24)	14.5 (25)	16.0 (34)	13.76	0.017
Han nationality	5.0 (18)	3.3 (12)	7.1 (27)	9.4 (36)	10.0 (39)	11.6 (71)	29.62	<0.001
Education level								
Junior high school or lower	9.7 (7)	12.8 (6)	12.1 (8)	18.9 (10)	22.4 (11)	8.6 (3)	6.16	0.291
Senior high school	5.4 (6)	4.0 (4)	9.6 (11)	12.1 (17)	14.6 (15)	20.0 (22)	19.22	0.002
College or higher	3.1 (6)	2.1 (5)	5.4 (12)	4.8 (10)	5.6 (14)	9.6 (47)	22.03	0.001
Monthly income (CNY)								
0	2.0 (1)	1.9 (1)	2.2 (1)	12.8 (5)	10.7 (6)	8.3 (7)	10.51	0.062^b^
≤2000	8.8 (11)	4.5 (4)	15.2 (15)	10.8 (7)	11.1 (3)	10.6 (5)	6.14	0.293
2001–3000	4.7 (4)	4.2 (4)	5.3 (5)	12.5 (14)	11.5 (9)	17.3 (17)	15.94	0.007
3001–4000	1.7 (1)	5.0 (3)	2.9 (2)	8.1 (7)	12.5 (9)	14.3 (16)	13.95	0.016
>4000	3.3 (2)	3.4 (3)	8.2 (8)	4.1 (4)	7.7 (13)	9.2 (27)	6.80	0.236
Self-perceived sexual orientation								
Homosexual	5.2 (12)	2.8 (7)	8.3 (22)	10 (26)	10.1 (25)	10.9 (49)	18.66	0.002
Bisexual	5.7 (6)	7.3 (8)	7.7 (9)	9.2 (11)	10.1 (12)	11.5 (17)	3.38	0.642
Uncertain	2.5 (1)	0.0 (0)	0.0 (0)	0.0 (0)	9.7 (3)	17.6 (6)	16.02	0.007^b^
Venues for meeting partners								
Bar, disco, tea house or club	3.6 (1)	16.1 (5)	4.0 (1)	11.1 (3)	13.6 (3)	18.2 (4)	5.57	0.350^b^
Bath house, sauna or massage	8.0 (2)	20.0 (3)	14.3 (2)	0.0 (0)	16.7 (1)	37.5 (3)	6.73	0.241^b^
Park, public toilet	3.5 (2)	4.0 (1)	0.0 (0)	16.7 (3)	22.5 (9)	0.0 (0)	16.68	0.005^b^
Internet	5.7 (14)	1.8 (5)	7.7 (23)	9.4 (29)	7.4 (21)	10.5 (59)	22.09	0.001
Other	0.0 (0)	2.4 (1)	11.4 (5)	5.4 (2)	11.8 (6)	17.6 (6)	10.97	0.052^b^
Number of male partners in the past 6 months								
1	4.3 (4)	2.4 (3)	6.6 (8)	7.3 (9)	8.8 (12)	9.2 (17)	7.33	0.197
2	2.5 (2)	3.3 (3)	7.3 (7)	6.1 (6)	9.3 (9)	8.8 (14)	6.35	0.274
3	1.4 (1)	4.5 (3)	7.9 (5)	16.7 (11)	8.3 (6)	11.6 (13)	12.52	0.028
4–9	5.8 (4)	2.6 (1)	10.0 (6)	11.8 (8)	15.0 (6)	18.1 (19)	10.19	0.070
≥10	16.7 (4)	17.2 (5)	20.0 (4)	7.1 (1)	11.1 (2)	25.0 (3)	2.24	0.815^b^
UAI^c^								
Yes	6.4 (15)	3.8 (8)	9.2 (22)	10.1 (25)	11.5 (26)	15.5 (56)	25.12	<0.001
No	2.7 (4)	4.0 (7)	5.5 (9)	7.9 (12)	8.0 (14)	5.9 (16)	6.48	0.262
UVI^c^								
Yes	3.4 (2)	2.0 (1)	7.8 (5)	11.5 (6)	10.0 (6)	11.3 (6)	7.11	0.213^b^
No	5.3 (17)	4.2 (14)	7.6 (26)	8.9 (31)	10.0 (34)	11.4 (66)	20.21	0.001
HIV test in the past 12 months								
Yes	3.2 (2)	2.6 (7)	6.2 (11)	8.5 (15)	7.6 (15)	10.4 (35)	16.54	0.005
No	5.4 (17)	7.2 (8)	8.8 (20)	10.0 (22)	12.7 (25)	12.5 (37)	12.76	0.026
STI symptoms in the past 12 months^c^								
Yes	2.4 (2)	8.2 (7)	14.8 (9)	10.9 (7)	10.5 (6)	18.4 (16)	13.17	0.022
No	5.8 (17)	2.7 (8)	6.4 (22)	8.9 (30)	9.9 (34)	10.3 (56)	21.04	0.001
Syphilis infection								
Yes	13.6 (9)	12.5 (4)	21.4 (6)	16.0 (4)	19.4 (7)	23.8 (5)	2.25	0.813
No	3.2 (10)	3.1 (11)	6.6 (25)	8.8 (33)	9.0 (33)	10.9 (67)	31.30	<0.001

a Pearson chi-square test.

b Likelihood ratio chi-square test.

c UAI, unprotected anal intercourse; UVI, unprotected vaginal intercourse; STI, sexually transmitted infection.

Factors associated with HIV infection were reported in Table 3. Those having UAI (OR = 1.80, 95% CI: 1.26–2.58), without Guangdong Province Hukou (OR = 2.02, 95% confidence interval (CI): 1.33–3.07, compared with those with Guangzhou local Hukou), having more than 10 partners (OR = 2.10, 95% CI: 1.14–3.87, compared with those having only 1 partner), and being syphilis positive (OR = 2.72, 95% CI: 1.74–4.26) were associated with higher risk for HIV infection. Having a college or higher education level (OR = 0.54, 95% CI: 0.34–0.85) and having HIV test in the past 12 months (OR = 0.73, 95% CI: 0.53–1.00) were associated with lower risk for HIV infection. After adjusting for demographic characteristics and key behaviors of the participants, the survey year remained an associated factor for HIV infection, indicating an increasing trend in HIV prevalence over years.

**Table 3:** Factors associated with HIV infection among MSM in Guangzhou, 2008–2013, by Logistic regression model.

Factor	B	Wald	*p*	OR (95% CI)
UAI^a^	0.59	10.41	0.001	1.80 (1.26–2.58)
Hukou (registered permanent residence)				
Guangzhou				1.00
Other city in Guangdong province	0.36	2.19	0.139	1.43 (0.89–2.29)
Outside Guangdong province	0.70	10.81	0.001	2.02 (1.33–3.07)
Education level				
Junior High school or lower				1.00
Senior high school	−0.03	0.01	0.905	0.97 (0.63–1.52)
College or higher	−0.61	7.07	0.008	0.54 (0.34–0.85)
Syphilis infection	1.00	19.33	<0.001	2.72 (1.74–4.26)
HIV test in the past 12 months	−0.32	3.84	0.050	0.73 (0.53–1.00)
Number of male partners in the past 6 months				
1				1.00
2	−0.21	0.93	0.336	0.81 (0.52–1.25)
3	0.08	0.11	0.738	1.08 (0.69–1.69)
4–9	0.33	2.12	0.145	1.39 (0.89–2.16)
≥10	0.74	5.67	0.017	2.10 (1.14–3.87)
Year				
2008				1.00
2009	0.41	1.08	0.299	1.50 (0.70–3.25)
2010	1.02	8.94	0.003	2.78 (1.42–5.44)
2011	1.20	12.62	<0.001	3.31 (1.71–6.41)
2012	1.37	16.27	<0.001	3.92 (2.02–7.60)
2013	1.80	31.14	<0.001	6.07 (3.22–11.43)

a UAI, unprotected anal intercourse.

UAI rates were high and stable in every single year, ranging from 54.5% to 62.0%. In Table 4, further analysis indicated the UAI among HIV-positive MSM was higher than that among HIV-negative MSM (71.0% versus 57.2%, p<0.001). UAI was generally higher among HIV positive cases in many subgroups especially those having a Hukou in other cities in Guangdong Province, having a college or higher education level, being syphilis positive, and having multiple male partners (Table 4).

**Table 4:** UAI rates of HIV-positive and HIV-negative MSM in Guangzhou stratified by the factors associated with HIV infection, 2008-2013.

Factor	UAI rate^a^ (%)		?^2^ value	*p* b
	HIV positive	HIV negative		
Total	71.0	57.2	15.41	<0.001
Age (years)				
<20	100.0	72.2	2.52	0.112^c^
20–29	74.8	57.6	14.75	<0.001
30–39	63.9	57.3	1.02	0.314
40–49	64.3	49.7	1.10	0.294
≥50	50.0	48.8	0.01	0.963^c^
Hukou (registered permanent residence)				
Guangzhou	66.7	56.2	1.67	0.197
Other city in Guangdong province	81.3	56.5	11.19	0.001
Outside Guangdong province	68.5	58.4	4.76	0.029
Education level				
Junior high school or lower	60.0	63.9	0.25	0.615
Senior high school	73.3	59.7	5.22	0.022
College or higher	74.5	55.0	13.62	<0.001
Syphilis infection				
Yes	88.6	57.8	11.82	0.001
No	67.6	57.2	7.38	0.007
HIV test in the past 12 months				
Yes	68.2	54.4	6.15	0.013
No	72.9	59.8	8.36	0.004
Number of male partners in the past 6 months				
1	69.8	58.5	2.61	0.106
2	78.0	62.3	4.09	0.043
3	74.4	66.7	0.94	0.333
4–9	88.6	69.3	7.12	0.008
≥10	78.9	72.4	0.35	0.557

a UAI, unprotected anal intercourse.

b Pearson chi-square test.

c Likelihood ratio chi-square test.

## Discussion

Results from 6 consecutive annual surveys indicated an increasing HIV prevalence among MSM in Guangzhou. The increase is a continuation of increasing trend since 2006 [21], coincides with the increase in the neighboring cities like Shenzhen [22], in similar Chinese metropolitan cities [9], [11], [23]–[25] and in the Asian region [17], [18], [26], [27]. Studies in similar metropolises such as Beijing and Shenyang also reported the high HIV incidence of 8.1 and 5.4 per 100 person-year, respectively [28], [29]. The increase of epidemic in China is different from the trend in San Francisco where HIV epidemic stabilized among MSM during the past decade in a context of decreasing HIV incidence and increased survival of HIV positive individuals due to ART [30].

The increase in HIV prevalence was also found in the most subgroups, by most demographic variables and related behaviors. This is consistent with our previous studies and the results from some studies in China [15], [31]–[32]. Stable prevalence over years was only found in some groups with high risk factors (having educational level of junior high school or lower, having 4 or more male partners, and syphilis infected) or protective factors (having less than 2 partners and no UAI), remaining high or low prevalence, respectively. In our study, after adjusting for years, UAI, migrant, multiple male partners, and syphilis infection were independent related risk factors of HIV infection, while higher educational level and taking an HIV test were protective factors.

Contrary to the trend of HIV prevalence, decreasing syphilis prevalence was found among MSM in Guangzhou from 2008 to 2013. Syphilis is a treatable disease which is passed on through sexual intercourse, therefore syphilis infection can be viewed as a marker for high-risk sexual practices, such as unprotected sex and/or multiple partners, and can also be controlled through screening and treatment. Previous study showed receiving HIV test is a protective factor of syphilis infection among MSM in Guangzhou [21], because when they take an HIV test, they can also receive free syphilis screening and referral services of treatment besides behavior intervention at the same time. Although there is limited data of syphilis screening and treatment among MSM in Guangzhou, our study showed the rate of having HIV test in the past 12 months has increased significantly during six years, arriving 53.4% in 2013, thus the rise of HIV test rate may contribute to control syphilis infection among these MSM. From 2008 to 2013, risk factors found associated with HIV infection decreased while protective factors increased across years. However, UAI, being consistently high and stable from 2008 to 2013, did not change from the level observed in our previous surveys and were not different from those observed in other studies in China [11], [15], [25], [31]–[33]. The consistently high level of UAI may explain the steady increase in HIV prevalence since UAI is the only biologically plausible but uncontrolled risk factor related to HIV during the past six years. HIV positive cases have higher UAI than those not infected yet. The finding, of 100% UAI among those aged below 20 years, is of a particular concern. This 100% UAI in the younger age group, together with high level of UAI in all subgroups, indicates an unhealthy culture environment and incorrect social norm in the MSM community. Social norms refer to expectations of acceptable behavior or attitudes within a community or peer group prescribed by the respective members [34]. The social norms of MSM community are to protect himself and his partners anytime having sex. Research among Chinese MSM found that stronger endorsement of positive social norms around condom use strongly predicted lower prevalence of HIV infection [10]. Therefore intervention program which focus on positive social norms and condom using may protect MSM from being infected with HIV.

This study has some limitations. Despite the snow-ball sampling applied, the majority of the sampled MSM were from venues or internet. This may bias the samples towards the relatively active MSM subgroups. Similar to other behavioral surveys, behavioral information was relied on self-reporting, recall bias and social desirability bias may exist. Information bias may also exist given the nature of sensitive sex-related questions. To address these possible biases, the same group of trained interviewers conducted all six surveys at the same site using an identical questionnaire. Consistency was ensured in the methodology and implementation of questionnaire interviews, biases may be towards one direction across years. The risk behaviors, such as UAI, reported by interviewees may be conservative due to social desirability bias. Despite these limitations, the present study is one of the few studies in China to examine the trend of the prevalence of HIV and behaviors among MSM for 6 consecutive years. In further research, cohort study will be conducted to measure incidences of HIV and syphilis among MSM and to modify cause inference.

In conclusion, HIV epidemic is expanding in Guangzhou. The persistently high UAI may have played a major role in the increasing trend of HIV prevalence. Targeted prevention program should be conducted among MSM who are migrants, low educational level, syphilis infected, or having multiple partners to encourage HIV test and change UAI behavior. The general high UAI calls for tailored intervention program to promote healthy culture and form a safe sex social norm in the MSM community.
